# miR-let-7c-5p and miR-149-5p inhibit proinflammatory cytokine production in osteoarthritis and rheumatoid arthritis synovial fibroblasts

**DOI:** 10.18632/aging.203201

**Published:** 2021-07-01

**Authors:** Yat-Yin Law, Wei-Fang Lee, Chin-Jung Hsu, Yen-You Lin, Chun-Hao Tsai, Chien-Chung Huang, Min-Huan Wu, Chih-Hsin Tang, Ju-Fang Liu

**Affiliations:** 1 Institute of Medicine, Chung Shan Medical University, Taichung, Taiwan; 2 Department of Orthopedics, Chung Shan Medical University Hospital, Taichung, Taiwan; 3 School of Dental Technology, College of Oral Medicine, Taipei Medical University, Taipei, Taiwan; 4 School of Chinese Medicine, China Medical University, Taichung, Taiwan; 5 Department of Orthopedic Surgery, China Medical University Hospital, Taichung, Taiwan; 6 School of Medicine, China Medical University, Taichung, Taiwan; 7 Department of Sports Medicine, College of Health Care, China Medical University, Taichung, Taiwan; 8 Division of Immunology and Rheumatology, Department of Internal Medicine, China Medical University Hospital, Taichung, Taiwan; 9 Bachelor of Science in Senior Wellness and Sports Science, Tunghai University, Taichung, Taiwan; 10 Tunghai University Sports Recreation and Health Management Degree Program, Tunghai University, Taichung, Taiwan; 11 Chinese Medicine Research Center, China Medical University, Taichung, Taiwan; 12 Department of Medical Laboratory Science and Biotechnology, College of Medical and Health Science, Asia University, Taichung, Taiwan; 13 School of Oral Hygiene, College of Oral Medicine, Taipei Medical University, Taipei, Taiwan

**Keywords:** miR-let-7c-5p, miR-149-5p, osteoarthritis, rheumatoid arthritis, inflammation

## Abstract

Osteoarthritis (OA) and rheumatoid arthritis (RA) are two of the most common types of arthritis. Both are characterized by the infiltration of a number of proinflammatory cytokines into the joint microenvironment. miRNAs play critical roles in the disease processes of arthritic disorders. However, little is known about the effects of miRNAs on critical inflammatory cytokine production with OA and RA progression. Here, we found higher levels of proinflammatory cytokines including interleukin 1 beta (IL-1β), interleukin 6 (IL-6) and tumor necrosis factor alpha (TNF-α) in human OA and RA synovial fibroblasts (SFs) compared with normal SFs. Searches of open-source microRNA (miRNA) software determined that miR-let-7c-5p and miR-149-5p interfere with IL-1β, IL-6 and TNF-α transcription; levels of all three proinflammatory cytokines were lower in human OA and RA patients compared with normal controls. Anti-inflammatory agents dexamethasone, celecoxib and indomethacin reduced proinflammatory cytokine production by promoting the expression of miR-let-7c-5p and miR-149-5p. Similarly, ibuprofen and methotrexate also enhanced miR-let-7c-5p and miR-149-5p expression in human SFs. The evidence suggests that increasing miR-let-7c-5p and miR-149-5p expression is a novel strategy for OA and RA.

## INTRODUCTION

Osteoarthritis (OA) and rheumatoid arthritis (RA) feature synovial inflammation and damage to articular cartilage, as well as pathological changes in subchondral bone [1]. Anti-inflammatories (NSAIDs and corticosteroids) are typically used to reduce ongoing inflammation and relieve the pain induced by arthritis [2, 3]. Patients with arthritis live with low-grade, chronic joint inflammation that perpetuates the release of inflammatory mediators, with ever-worsening damage to cartilage, bone and synovium [4, 5].

The activities of proinflammatory cytokines interleukin 1 beta (IL-1β), IL-6 and tumor necrosis factor alpha (TNF-α) contribute to the pathogenesis of arthritis by promoting proteolytic enzyme activity that damages the cartilage extracellular matrix [6, 7]. IL-1β, IL-6 and TNF-α levels in human arthritic serum and synovial fluid are higher than those of healthy controls and have been targeted by therapies including the IL-1β inhibitor canakinumab, the IL-6 inhibitor tocilizumab, and the TNF-α inhibitor infliximab [8]. Reducing proinflammatory cytokine activity has shown merit as a therapeutic strategy to reduce arthritis progression [6].

The progression of arthritis disease is regulated by several microRNAs (miRNAs), including miR-92a, miR-129-3p, miR-141-3p and miR-199a-5p [9–12], while the proinflammatory mediators IL-1β, IL-6, TNF-α and matrix metalloproteinases (MMPs) account for histological changes that occur with arthritis [13–15]. However, how miRNAs might regulate the progression of OA and RA remains unclear. We describe finding higher levels of proinflammatory cytokines in OA synovial fibroblasts (OASFs) and RASFs than in normal SFs (NSFs), while miR-let-7c-5p and miR-149-5p were associated with reductions in proinflammatory cytokine expression. Treatment of OASFs and RASFs with anti-inflammatory agents enhanced levels of miR-let-7c-5p and miR-149-5p expression and reduced proinflammatory responses. Thus, finding ways to promote the expression of these miRNAs may be of therapeutic benefit for patients with OA or RA.

## RESULTS

### Higher levels of proinflammatory cytokines in OA and RA patients

**Figure 1:**
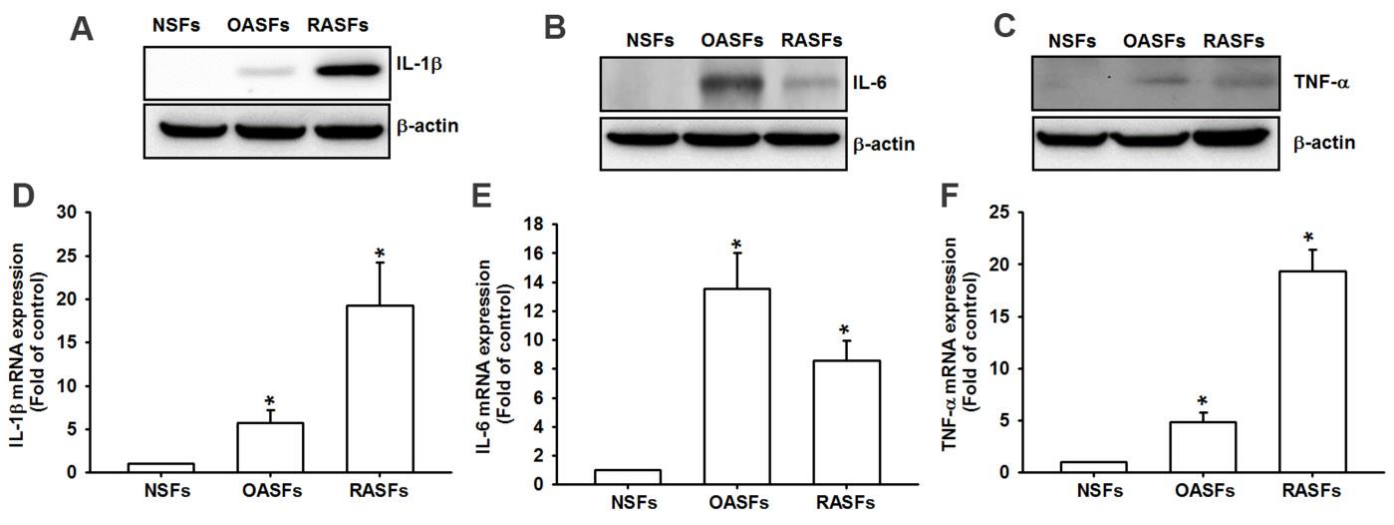
**Higher levels of proinflammatory cytokines in OA and RA synovial fibroblasts.** Levels of IL-1β, IL-6 and TNF-α protein (**A**–**C**) and mRNA expression (**D**–**F**) in NSFs, OASFs and RASFs were examined by Western blot and qPCR assays.

Similarly to previous research [8], we found markedly higher levels of IL-1β, IL-6 and TNF-α protein and mRNA in OASFs and RASFs than in NSFs (Figure 1), indicating that these cytokines regulate OA and RA progression.

### miR-let-7c-5p and miR-149-5p inhibit proinflammatory cytokine expression

**Figure 2:**
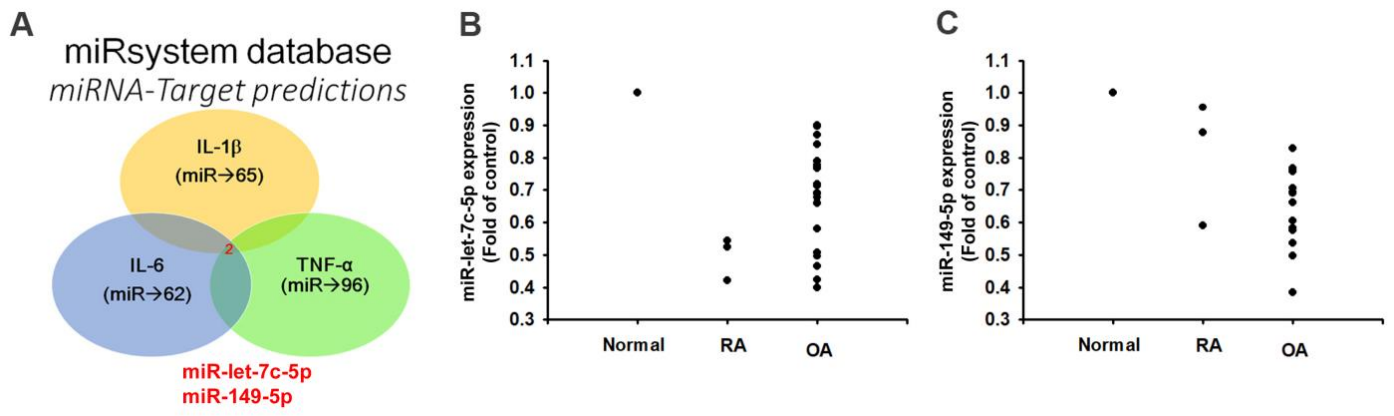
**Lower levels of miR-let-7c-5p and miR-149-5p expression in OA and RA patients compared with normal controls.** (**A**) Open-source software (miRWalk, miRanda, and TargetScan) sought to identify miRNAs that could possibly interfere with IL-1β, IL-6 and TNF-α transcription. (**B**, **C**) Levels of miR-let-7c-5p and miR-149-5p expression in normal controls (n=5), OA (n=3) and RA (n=12) patients were examined by qPCR.

**Figure 3:**
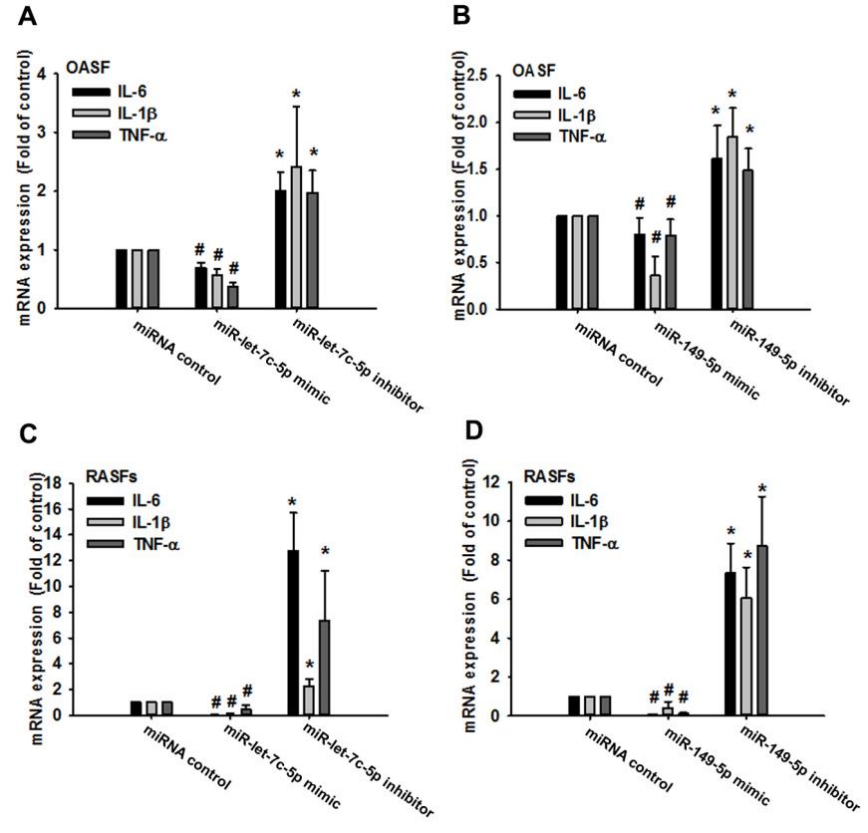
**MiR-let-7c-5p and miR-149-5p regulate IL-1β, IL-6 and TNF-α expression in OASFs and RASFs.** After transfecting OASFs (**A**, **B**) and RASFs (**C**, **D**) with the miR-let-7c-5p and miR-149-5p mimics or their respective inhibitors, IL-1β, IL-6 and TNF-α expression was examined by qPCR.

Records from the miRWalk, miRanda, and TargetScan online tools identified that 3’-UTRs of IL-1β, IL-6 and TNF-α mRNAs harbor potential binding sites for miR-let-7c-5p and miR-149-5p (Figure 2A). Levels of both miRNAs were lower in OA and RA patients than in normal controls (Figure 2B, 2C), indicating that these miRNAs are negatively correlated with the expression of IL-1β, IL-6 and TNF-α. Moreover, levels of all three cytokines were reduced in OASFs and RASFs transfected with the respective mimics of miR-let-7c-5p and miR-149-5p, whereas transfection with the respective inhibitors had the opposite effect (Figure 3), which suggests that miR-let-7c-5p and miR-149-5p negatively control the production of IL-1β, IL-6 and TNF-α.

### Anti-inflammatory agents reduce proinflammatory cytokine expression by increasing miR-let-7c-5p and miR-149-5p synthesis

**Figure 4:**
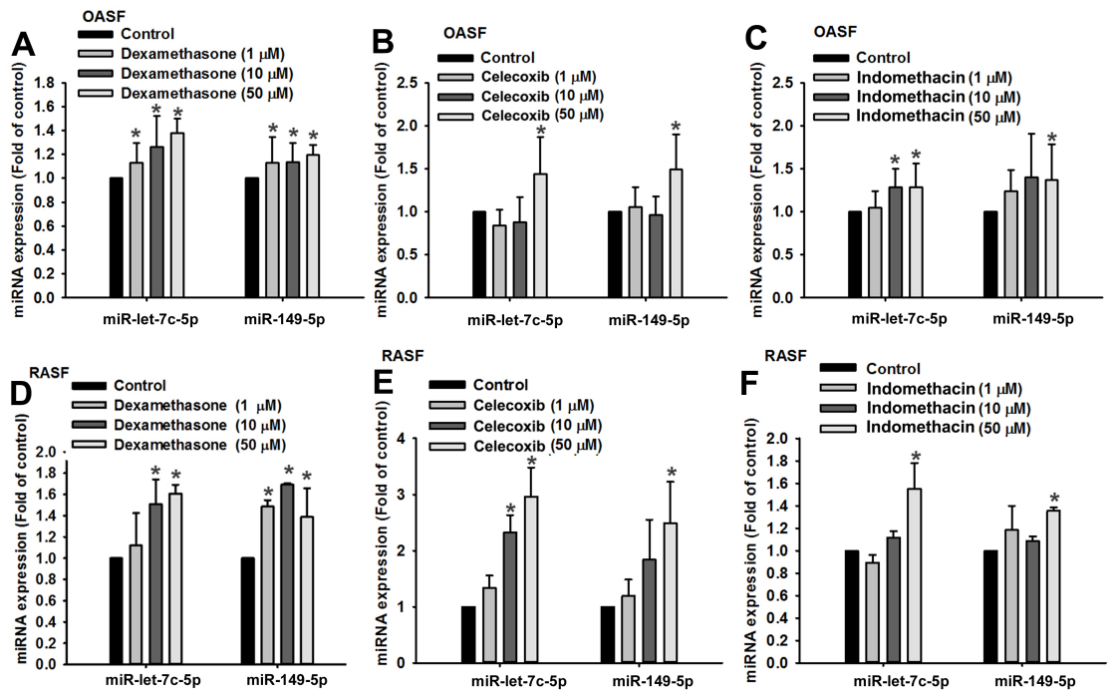
**Anti-inflammatory agents upregulate miR-let-7c-5p and miR-149-5p expression.** OASFs (**A**–**C**) and RASFs (**D**–**F**) were treated with dexamethasone, celecoxib, or indomethacin (1–50 μM), then subjected to qPCR quantification of miR-let-7c-5p and miR-149-5p expression.

**Figure 5:**
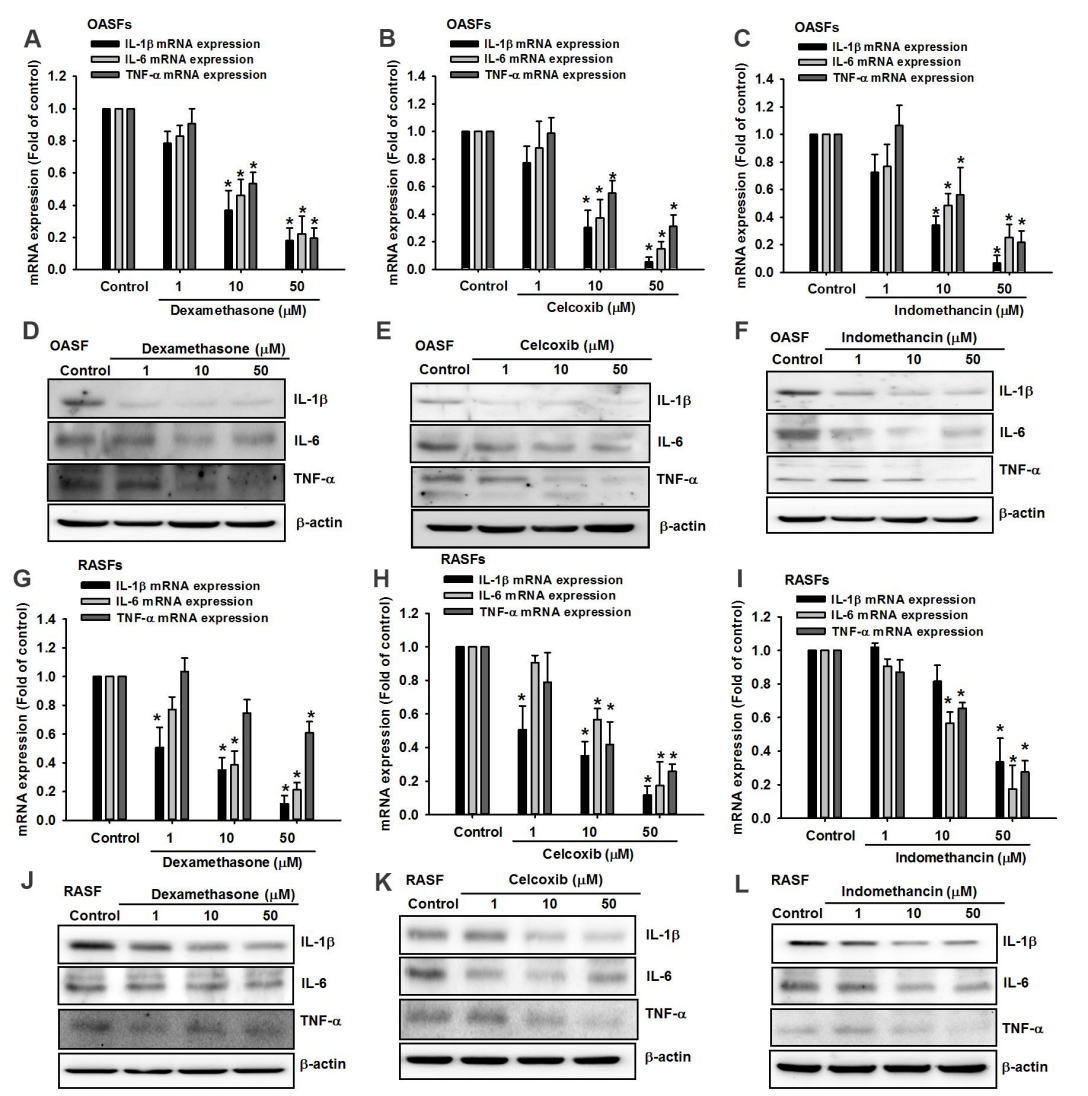
**Anti-inflammatory agents reduce proinflammatory cytokine production in OASFs and RASFs.** After treating OASFs (**A**–**F**) and RASFs (**G**–**L**) with dexamethasone, celecoxib, or indomethacin (1–50 μM), Western blot and qPCR assays quantified the levels of IL-1β, IL-6 and TNF-α expression.

**Figure 6:**
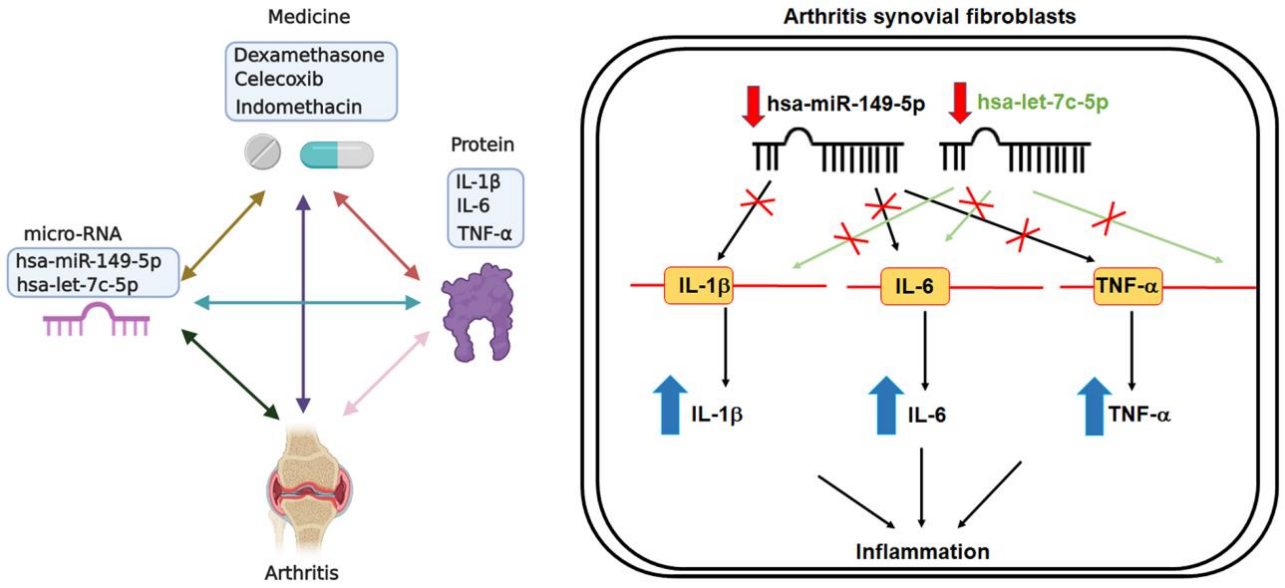
**Schematic diagram summarizes the effects of miR-let-7c-5p and miR-149-5p in OA ad RA.** Treatment of OASFs and RASFs with anti-inflammatory agents upregulates levels of miR-let-7c-5p and miR-149-5p expression, leading to decreases in the expression of proinflammatory cytokines (IL-1β, IL-6 and TNF-α). Thus, enhancing miR-let-7c-5p and miR-149-5p expression may be a novel therapeutic avenue for halting the progression of OA and RA disease.

Dexamethasone (a corticosteroid), celecoxib (a selective cyclooxygenase-2 inhibitor) and indomethacin (an NSAID) are all anti-inflammatory agents that are used to treat arthritis [16]. In this study, treatment of OASFs and RASFs with dexamethasone, celecoxib, or indomethacin facilitated the synthesis of both miR-let-7c-5p and miR-149-5p (Figure 4) and concentration-dependently reduced protein and mRNA levels of IL-1β, IL-6 and TNF-α (Figure 5). Thus, these anti-inflammatory agents reduce proinflammatory cytokine production in human synovial fibroblasts by enhancing miR-let-7c-5p and miR-149-5p expression (Figure 6). Similarly, ibuprofen (another commonly used NSAID) and methotrexate (considered to be the gold standard disease-modifying antirheumatic drug [DMARD]), also enhanced the expression of these miRNAs in human synovial fibroblasts (Supplementary Figure 1).

## DISCUSSION

OA and RA are well known for their major characteristics of synovial inflammation and joint destruction [17–19]. OASFs and RASFs in the joint microenvironment are important contributors to the disease processes, producing proinflammatory cytokines that lead to cartilage degradation and bone breakdown [20]. Several commercially available arthritis therapeutics target these proinflammatory cytokines. Amongst the many reports of different associations of human miRNAs and particular diseases, evidence attests to the efficiency of miRNA binding with their target regions and post-transcriptional regulation of target protein expression [21, 22]. However, little is known about the effect of miRNAs on critical inflammatory cytokine production and OA and RA progression. Our study results describe how OASFs and RASFs contain higher levels of proinflammatory cytokines compared with NSF levels. We also found that miR-let-7c-5p and miR-149-5p negatively regulate levels of IL-1β, IL-6 and TNF-α expression. The therapeutic effects of anti-inflammatory agents used in this study were associated with their inhibition of miR-let-7c-5p and miR-149-5p expression, indicating that modulation of these miRNAs may be a novel strategy for reducing OA and RA inflammation.

MiRNAs post-transcriptionally regulate gene expression [23]. During OA and RA progression, aberrant miRNA expression mediates inflammatory pathway signaling [24, 25]. Our examination of open-source database records identified that miR-let-7c-5p and miR-149-5p potentially interfere with the transcription of IL-1β, IL-6 and TNF-α. The levels of both miRNAs were lower in OA and RA patients than in normal controls, indicating negative correlations between miR-let-7c-5p and miR-149-5p and proinflammatory cytokine expression. There was no evidence of sequence similarity between these miRNAs. Other evidence has shown that miR-let-7c-5p inhibits the proliferation and migration of cervical carcinoma cells [26] and negatively regulates NLRC5 protein expression in ethanol-induced hepatic injury [27]. Interestingly, miR-149-5p is capable of inhibiting the growth of gastric cancer, cholangiocarcinoma and glioblastoma [28–30]. MiR-149-5p can also inhibit M1 macrophage-associated inflammation in experimental abdominal aortic aneurysm formation [31]. In this study, we enhanced miR-let-7c-5p and miR-149-5p levels in OASFs and RASFs by transfecting them with their respective miRNA mimics, which also reduced IL-1β, IL-6 and TNF-α production. In contrast, transfection of OASFs and RASFs with miR-let-7c-5p and miR-149-5p inhibitors downregulated the levels of both miRNAs. Thus, our evidence has identified novel anti-inflammatory functions in association with miR-let-7c-5p and miR-149-5p. Numerous signaling pathways, including the MAPK, PI3K/Akt and PKC pathways, control miRNA synthesis during the progression of arthritis disease [9–12]. We did not include the upstream molecules of miR-let-7c-5p and miR-149-5p in this investigation. Further research is needed to determine whether their expression is regulated by the same signaling cascades.

Anti-inflammatory agents (e.g., corticosteroids, COX-2 selective inhibitors and NSAIDs), are well recognized for their modulation of inflammatory responses in different inflammatory diseases, including arthritis [16]. Our study results confirm that dexamethasone, celecoxib and indomethacin effectively lower the synthesis of arthritic inflammatory cytokines, including IL-1β, IL-6 and TNF-α, in both OASFs and RASFs, by upregulating miR-let-7c-5p and miR-149-5p expression. Our genetic and pharmacologic investigations suggest that enhancing the levels of these miRNAs may be a novel avenue for treating OA and RA.

## MATERIALS AND METHODS

### Materials

Antibodies for IL-1β, IL-6 and TNF-α were obtained from GeneTex International Corporation. Lipofectamine^®^ 2000 and Trizol^®^ were acquired from Life Technologies. The miRNA mimic, inhibitor and negative control and Dharmafect1 were purchased from Dharmacon. β-Actin antibody and all other chemicals not already mentioned were acquired from Sigma-Aldrich.

### Cell preparation

Human NSFs (primary fibroblast-like synoviocytes; 408-05A) were obtained from Cell Applications, Inc. (San Diego, CA, USA). Human RASFs were purchased from the Riken Cell Bank (Ibaraki, Japan). Human OASFs were isolated from synovial tissues of 10 OA patients by collagenase treatment, using previously detailed procedures [32]. All cells were maintained in DMEM (containing 10% FBS and antibiotics) in a 5% CO_2_ incubator (at 37° C).

### Human synovial tissues

Study approval was granted by the Institutional Review Board of China Medical University Hospital (Taichung, Taiwan) and all patients provided written informed consent before participating in the study. Synovial tissue samples were obtained from patients undergoing total knee arthroplasty for OA or RA and also from those undergoing arthroscopic procedures for trauma/joint derangement (healthy controls).

### Western blot

SDS-PAGE was used to resolve the extracted proteins, which were transferred to PVDF membranes, as described in our previous publications [33–35]. Membranes were blocked for 1 h with PBST containing 4% non-fat milk, then treated with antibodies targeting IL-1β, IL-6 and TNF-α for 1 h, before being incubated for 1 h with HRP-conjugated secondary antibodies. We visualized the blot membranes using a Fujifilm LAS-3000 imaging system.

### Quantitative real-time PCR

All RNA was collected from SFs using TRIzol™ Reagent. We generated cDNA using an Invitrogen reverse transcription kit. qPCR analysis was conducted with the Taqman^®^ One-Step RT-PCR Master Mix. Mir-X™ miRNA First-Strand Synthesis and the SYBR^®^ RT-PCR kit were used for reverse transcription of miRNA. Analysis was carried out according to a previous protocol [36–38].

### Transfection

Synthetic miRNA mimics and inhibitors (10 nM) were transfected into OASFs and RASFs following the Dharmafect1 transfection protocol. After 24 h of transfection, inflammatory cytokine expression was examined by qPCR.

### Statistics

All values are presented as the mean ± standard deviation of 5 independent experiments. Differences between two experimental groups were assessed for significance using the Student’s *t*-test and considered to be significant if the *p* value was < 0.05.

## Supplementary Material

Supplementary Figure 1
