# Supplementary material for: miR-let-7c-5p and miR-149-5p inhibit proinflammatory cytokine production in osteoarthritis and rheumatoid arthritis synovial fibroblasts
Source: Aging (Albany NY). 2021 Jul 1;13(13):17227–36. doi: 10.18632/aging.203201 (PMC8312412; doi:10.18632/aging.203201)
Supplement: Supplementary Figure 1 [file aging-13-203201-s001.pdf]

SUPPLEMENTARY FIGURE

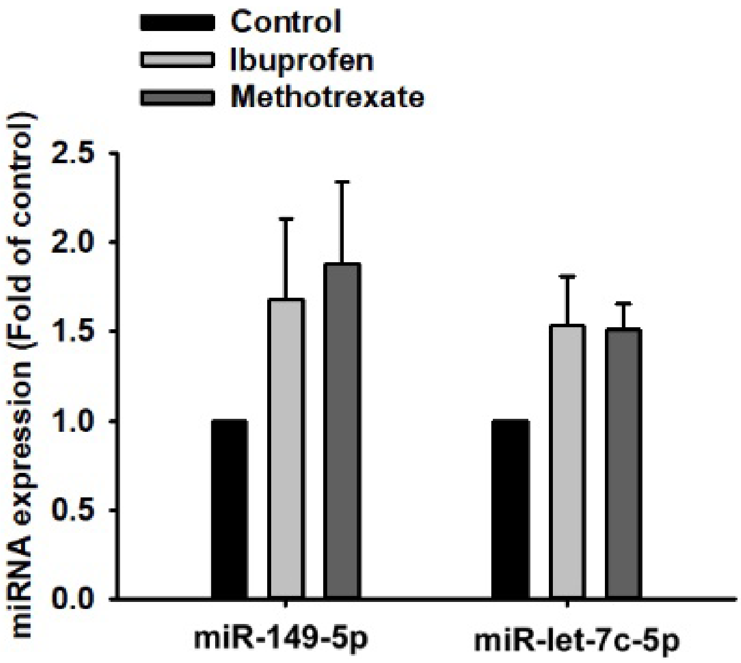

Supplementary Figure 1. Ibuprofen and methotrexate upregulate miR-let-7c-5p and miR-149-5p expression. Synovial fibroblasts were treated with ibuprofen and methotrexate, then subjected to qPCR quantification of miR-let-7c-5p and miR-149-5p expression.
